# First insights into the phylogenetic diversity of *Mycobacterium tuberculosis* in Kuwait and evaluation of REBA MTB-MDR assay for rapid detection of MDR-TB

**DOI:** 10.1371/journal.pone.0276487

**Published:** 2022-10-20

**Authors:** Noura M. Al-Mutairi, Suhail Ahmad, Eiman Mokaddas, Sahal Al-Hajoj

**Affiliations:** 1 Department of Microbiology, Faculty of Medicine, Kuwait University, Safat, Kuwait; 2 Kuwait National TB Control Laboratory, Shuwaikh, Kuwait; 3 Department of Infection and Immunity, Mycobacteriology Research Section, King Faisal Special Hospital and Research Center (KFSH & RC), Riyadh, Saudi Arabia; Indian Institute of Science Education and Research Bhopal, INDIA

## Abstract

Early detection of *Mycobacterium tuberculosis* (Mtb) in clinical specimens, its susceptibility to anti-TB drugs and disruption of infection transmission to new hosts are essential components for global tuberculosis (TB) control efforts. This study investigated major Mtb genotypes circulating in Kuwait and evaluated the performance of REBA MTB-MDR (REBA) test in comparison to GenoType MTBDR*plus* (gMTBDR^+^) assay for rapid detection of resistance of Mtb to isoniazid and rifampicin (MDR-TB). *M*. *tuberculosis* isolates (n = 256) originating predominantly from expatriate patients during a 6-month period were tested by spoligotyping and a dendrogram was created by UPGMA using MIRU-VNTRplus software. Phenotypic drug susceptibility testing (DST) was performed by MGIT 960 system. Genotypic DST for isoniazid and rifampicin was done by REBA and gMTBDR^+^ assays. Spoligotyping assigned 188 (73.4%) isolates to specific spoligotype international type (SIT) while 68 isolates exhibited orphan patterns. All major *M*. *tuberculosis* lineages were detected and EAI, CAS and Beijing families were predominant. Phylogenetic tree showed 131 patterns with 105 isolates exhibiting a unique pattern while 151 isolates clustered in 26 patterns. Fifteen isolates were resistant to one/more drugs. REBA and gMTBDR^+^ detected isoniazid resistance in 11/12 and 10/12 and rifampicin resistance in 4/5 and 4/5 resistant isolates, respectively. The diversity of SIT patterns are highly suggestive of infection of most expatriate patients with unique Mtb strains, likely acquired in their native countries before their arrival in Kuwait. Both, REBA and gMTBDR^+^ assays performed similarly for detection of resistance of Mtb to isoniazid and rifampicin for rapid detection of MDR-TB.

## Introduction

Tuberculosis (TB) is a major infectious disease caused by the obligate human pathogen, *Mycobacterium tuberculosis* [1]. According to the annual global health surveys published by World Health Organization (WHO), nearly 10 million active TB cases and about 1.4 million deaths occurred in 2019 [2]. Nearly 66% of the global TB cases occurred in only eight (India, Indonesia, China, the Philippines, Pakistan, Nigeria, Bangladesh and South Africa) countries [2]. Around 465000 people in 2019 developed TB that was resistant to rifampicin (RR-TB), and of these, about 363000 (78%) individuals had multidrug-resistant (MDR)-TB (defined as infection with *M*. *tuberculosis* resistant at least to the two most effective first-line drugs; rifampicin, RIF and isoniazid, INH) [2]. Moreover, nearly 6.2% of MDR-TB cases were estimated to have extensively drug resistant (XDR)-TB (MDR-TB strains which are additionally resistant to a fluoroquinolone and an injectable agent; amikacin, kanamycin, or capreomycin; pre-2021 definition) [2]. Due to lower cure rate for RR-TB/MDR-TB/XDR-TB [3], increasing incidence of drug-resistant TB (DR-TB) is a serious threat to global TB control efforts [2, 4, 5]. Epidemiological studies have shown that in high-TB-burden countries, active TB disease cases usually occur as a result of recent infection or reinfection while in low-TB-incidence countries, most active disease cases occur as a result of reactivation of latent infection acquired few to several years earlier [2, 5–8].

Molecular fingerprinting of *M*. *tuberculosis* is performed to determine TB transmission and epidemiology for better control of active TB disease in a population. Spoligotyping is a relatively simple typing method which detects polymorphisms at direct repeat (DR) locus belonging to the clustered regularly interspaced short palindromic repeats (CRISPRs) family of repetitive DNA [9, 10]. Although spoligotypes are usually less discriminatory for *M*. *tuberculosis* strains in high TB burden countries experiencing active disease transmission, they are highly discriminatory for low TB incidence settings, particularly for countries with large and diverse expatriate population [11–13]. Additional fingerprinting data are obtained by mycobacterial interspersed repetitive units (MIRUs)-variable number tandem repeat (VNTR) typing or other methods such as restriction fragment length polymorphism (RFLP) or whole genome sequencing (WGS) [10, 14–16].

The WHO has endorsed GenoType MTBDR*plus* line probe assay (gMTBDR^+^) as one of the molecular tests for the rapid diagnosis of MDR-TB due to slow turn-around time of the phenotypic drug susceptibility testing (DST) of *M*. *tuberculosis* [17, 18]. REBA MTB-MDR (REBA) is another line probe assay developed by YD diagnostics for simultaneous detection of resistance of *M*. *tuberculosis* to INH and RIF (MDR-TB) which, due to its lower cost, is also suitable for resource-poor settings [19]. The REBA test has been evaluated for sensitive and specific detection of MDR-TB previously and its performance has also been compared with gMTBDR^+^ assay [19, 20]. However, the performance of REBA has not been evaluated in different geographical settings.

Kuwait, a small Arabian Gulf country in the Middle East, is estimated to have nearly 800 TB cases/year with an incidence rate of 23 cases per 100,000 population [4]. The expatriates account for nearly 70% of the total population of Kuwait. Although all expatriates are screened for the absence of active TB disease at the time of their entry into Kuwait, >80% of all TB cases and >90% of DR-TB and MDR-TB cases occur among expatriate patients, mainly originating from TB endemic countries of South Asia (Bangladesh, India, Nepal, Pakistan, Sri Lanka etc.), Southeast Asia (mainly Philippines and Indonesia) and Africa (Egypt, Ethiopia, Nigeria etc.) [21–24]. Although TB is currently not a major public health problem in Kuwait, recent increase in the number of expatriates from areas endemic for TB strongly advocates the need for surveillance programs [4, 25]. We have previously used spoligotyping for determining strain relatedness among MDR-TB strains in Kuwait and showed considerable genotypic heterogeneity among the isolates [22]. However, all isolates in that study were MDR-TB strains which were collected over a 15-year period [22]. In this study, we investigated the prevalence of different *M*. *tuberculosis* genotypes among all available cultured isolates collected during a 6-month period in Kuwait. Conventional (phenotypic) DST of *M*. *tuberculosis* isolates against four (streptomycin, STR, INH, RIF and ethambutol, EMB) anti-TB drugs was done by mycobacteria growth indicator tube (MGIT) 960 system. Performance of REBA and gMTBDR^+^ assays in detecting resistance to INH and RIF among phenotypically drug-resistant *M*. *tuberculosis* isolates was also studied.

## Materials and methods

### *M*. *tuberculosis* isolates

A total of 256 *M*. *tuberculosis* isolates collected from 256 patients during a six-month period (October 2017 to March 2018) from Kuwait National TB Control Laboratory (KNTCL) and representing all available isolates collected during this period were used. These isolates were obtained from 209 pulmonary and 47 extrapulmonary samples (details are provided in S1 Table). The study was approved by the Health Sciences Center Ethical Committee, Kuwait University (Approval no. VDR/EC/3762) and all experimental procedures and investigations were performed in accordance with their guidelines and regulations. As the study did not involve direct contact with patients and the results are reported on deidentified samples without revealing patient identity, the need for informed consent was waived by the Health Sciences Center Ethical Committee.

### Cultivation of *M*. *tuberculosis*, identification and drug susceptibility testing

Non-sterile clinical samples were processed by the standard *N*-acetyl-L-cysteine and sodium hydroxide (NALC/NaOH) method while sterile specimens were processed directly for the isolation of *M*. *tuberculosis* [26]. All samples were cultured on solid (Lowenstein-Jensen) and MGIT 960 system media according to the manufacturer’s instructions (Becton Dickinson, Sparks, MD, USA) and as described previously [23, 26]. Genomic DNA was prepared from MGIT culture tubes by using Chelex-100 as described previously [27]. Briefly, 1 ml of MGIT 960 system culture was heated with 20 mg of Chelex-100 at 95°C for 10 min, the contents were centrifuged at 12,000 X g for 15 min, the supernatant was removed and was used as the source of genomic DNA. All MGIT cultures were tested for the presence of *M*. *tuberculosis* complex DNA by AccuProbe DNA probe assay and by an in-house multiplex PCR assay that discriminates *M*. *tuberculosis* complex members from non-tuberculous mycobacteria, performed as described previously [28]. All *M*. *tuberculosis* isolates were subjected to phenotypic DST against RIF, INH, EMB and STR by using SIRE drug kit, according to the manufacturer’s recommendations and as described in detail previously [23, 26]. The final drug concentrations were 1.0 mg/L for RIF, 0.1 mg/L for INH, 5.0 mg/L for EMB and 1.0 mg/L for STR. The DST results were interpreted and reported automatically by the MGIT system using predefined algorithms that compare the growth of *M*. *tuberculosis* in the drug-containing tube with the growth in the drug-free control tube [23].

### Spoligotyping

The spoligotyping method comprises two steps; amplification of the 43 DNA spacers by PCR followed by hybridization of PCR-amplified spacer DNA and detection of hybridized products. All 256 *M*. *tuberculosis* isolates were subjected to spoligotyping using SPOLIGO TB kit (Mapmygenome Co., Hyderabad, India) and genomic DNA prepared from MGIT 960 system cultures as described above. The SPOLIGO TB kit was used according to the instructions supplied with the kit and the results were interpreted as described previously [9, 22]. Briefly, the 43 spacers within direct repeat region were amplified using primers Dra and biotin-labeled Drb supplied with the kit, the biotinylated amplicons were hybridized with 43 oligonucleotides bound covalently in a line pattern on Spoligo-membrane provided with the kit under strictly controlled conditions in a mini-blotter apparatus according to the manufacturer instructions.

The hybridization signals were detected by enhanced chemiluminescence (ECL) detection system (West Pico PLUS Chemiluminescent Substrate; Thermo Scientific, Carlsbad, CA, USA) according to the manufactures instructions by incubating the membrane with working solution prepared by mixing equal parts of the Stable Peroxide Solution and the Luminol/Enhancer Solution (provided with the kit) for 10 min at room temperature. As a result, the peroxidase coupled to streptavidine causes emission of light, which is detected by UVP machine (Analytikjena, An Endress + Hauser Company, Cambridge, UK). The detected bands were converted to 43 binary codes which were used for assignment of phylogenetic lineages according to SITVIT2 database (http://www.pasteur-guadeloupe.fr:8081/SITVIT2/). *M*. *tuberculosis* H37Rv and *M*. *bovis* BCG (Pasteur strain) were used as control strains.

The spoligotype-based dendrogram was generated by unweighted pair group method with arithmetic mean (UPGMA) using MIRU-VNTR plus database. The minimum spanning tree was generated using BioNumerics v7.6 software (Applied Maths, Saint-Martens-Latem, Belgium). The discriminatory power was determined by calculating the Hunter Gaston Discriminatory Index (HGDI) using Discriminatory Power Calculator (http://insilico.ehu.es/mini_tools/discriminatory_power). Non-clustered isolates had unique spoligotype patterns that were not identical to any of the Mtb isolate included in the study. A cluster was defined when two or more patient isolates shared the same spoligotype pattern. The “Unknown” indicates spoligotype patterns with signatures reported in SITVIT2 database that do not belong to any of the major *M*. *tuberculosis* lineages. An spoligotype pattern not described in SITVIT2 database was termed as ’orphan’ pattern [29]. Clustering rate was calculated using the formula (nc-c)/n * 100 where the nc: is the total number of clustered cases, c: is the number of clusters, n: is the total number of cases in the study [30].

### MolecuTech REBA MTB-MDR and GenoType MTBDR*plus* assays for drug resistant *M*. *tuberculosis* isolates

Fourteen drug-susceptible and all DR-TB isolates were tested by REBA line probe assay which simultaneously detects RIF and INH resistance of *M*. *tuberculosis* strains based on multiplex PCR amplification of *rpoB*, *katG*, the *inhA* promoter region and the *oxyR-ahpC* intergenic region by using the REBA MTB-MDR primer mixture. The biotinylated amplicons of multiplex PCR were hybridized to probes impregnated on nitrocellulose paper strips provided with the kit under strictly controlled conditions. Hybridized amplicons were detected by addition of streptavidin-alkaline phosphatase conjugate followed by color development with chromogenic substrate. The REBA assay was performed and the results were interpreted according to manufacturer instructions (YD Diagnostics, Gyeonggi-do, Republic of Korea) and as described previously [19].

The REBA test strip contains 21 probes including a mycobacterial genus-specific probe (Myc), 1 probe specific for *M*. *tuberculosis* complex (MTB) and 19 probes for RIF and INH resistance detection [19]. For RIF resistance, the test strip contains 5 probes (*rpoB*WT1-*rpoB*WT5) for detecting wild-type *rpoB* sequence and 3 probes (*rpoB*MT1 for S450L, *rpoB*MT2 for L452P and *rpoB*MT3 for D435V; *M*. *tuberculosis* codon numbering) for detection of 3 specific *rpoB* mutations. For INH resistance, the test strip contains 1 probe each for detection of wild-type (*katG*WT) and S315T mutation (*katG*MT) at *katG* codon 315, 2 probes (*inhA*WT1 and *inhA*WT2) for detecting wild-type and 2 probes (*inhA*MT1 for -15 C/T and *inhA*MT2 for -8 T/C) for detection of 2 specific mutations in *inhA* promoter region as well as 5 probes (*ahpC*WT1-*ahpC*WT5) for detecting wild-type *oxyR*-*ahpC* intergenic region sequence [19]. A signal with all wild-type probes indicates absence of a mutation. The presence of mutations suggesting resistance is indicated either by lack of hybridization to the respective wild-type probe(s) or by lack of hybridization to the wild-type probe(s) together with hybridization to one or more of the mutant probes [19]. Water was included instead of DNA as negative control.

The results of REBA were compared with gMTBDR^+^ line probe assay which was performed according to manufacturer (Hain Lifescience GmbH, Nehren, Germany) instructions and as described previously [31]. The gMTBDR^+^ test strip contains 26 probes including an amplification control (AC), 1 probe (TUB) specific for *M*. *tuberculosis* complex and 24 probes for RIF and INH resistance detection. For RIF resistance, the test strip contains 1 probe (*rpoB*) for detection of *rpoB* gene sequence, 8 probes (*rpoB*WT1-*rpoB*WT8) for detecting wild-type *rpoB* sequence and 4 probes (*rpoB*MUT1 for D441V, *rpoB*MUT2A for H451Y, *rpoB*MUT2B for H451D and *rpoB*MUT3 for S456L) for detection of 4 specific *rpoB* mutations. For INH resistance, the test strip contains 4 probes for *katG* and 7 probes for *inhA* promoter region. The *katG* probes include 1 probe (*katG*) for detection of *katG* gene sequence, 1 probe for detection of wild-type (*katG*WT) and 2 probes (*katG*MUT1, AGC to ACC and *katG*MUT2, AGC to ACA) for S315T mutation at *katG* codon 315. The *inhA* probes include 1 probe (*inhA*) for detection of *inhA* promoter region, 2 probes (*inhA*WT1 and *inhA*WT2) for detecting wild-type sequence and 4 probes (*inhA*MUT1 for -15 C/T, *inhA*MUT2 for -16 A/G, *inhA*MUT2A for -8 T/C and *inhA*MUT2B for -8 T/A) for detection of 4 specific mutations in *inhA* promoter region. A signal with all wild-type probes indicates absence of a mutation. The presence of mutations suggesting resistance is indicated either by lack of hybridization to the respective wild-type probe(s) or by lack of hybridization to the wild-type probe(s) together with hybridization to one or more of the mutant probes, as explained in the manufacturer instructions and described previously [31].

Lack of hybridization with a wild-type probe in REBA and gMTBDR^+^ tests could also result due to a non-synonymous mutation in the amplified gene fragment for various genes implicated in drug resistance [17]. To resolve the ambiguous or discordant results, PCR sequencing of the hot-spot and N-terminal regions of *rpoB* gene was performed to identify specific mutations in phenotypically RIF-resistant *M*. *tuberculosis* isolates that hybridized to all wild-type probes or lacked a signal with a wild-type probe without hybridization with a mutant probe in REBA and/or gMTBDR^+^ assay, as described previously [31, 32].

The hot spot region of *rpoB* gene was amplified with primers HSRF 5’-GACGACATCGACCACTTCGGCAAC-3’ and HSRR 5’-GAACGGGTTGACCCGCGCGTACA-3’ and 426 bp amplicons were purified and sequenced with internal primers; HSRFS, 5’- GCATGTCGCGGATGGAGCGGGT- 3’ and HSRRS, 5’- GCGTACACCGACAGCGAGCCGA- 3’. The N-terminal fragment was amplified by using NTF 5’-CGACGAGTGCAAAGACAAGGAC-3’ and NTR 5’-GACGGTGTCGCGCTTGTCGAC-3’ primers and the 310 bp amplicon was purified and sequenced by using the same amplification primers and the sequencing protocol described previously [31, 32]. PCR sequencing was also performed to identify specific mutation(s) in the *oxyR*-*ahpC* intergenic region in *M*. *tuberculosis* isolates that were detected by lack of signal with *ahpC* wild-type probe. The *oxyR*-*ahpC* intergenic region was amplified with primers AHPCF 5’-AGCGTCTGGTCGCGTAGGCAGTG-3’ and AHPCR 5’-AGCAGCCCGGCGACTACTTCACC-3’ and the 470 bp amplicons were sequenced with internal forward primer AHPCFS 5’-CAGTCACAACAAAGTCAGCTCTGACA-3’ or AHPCR primer. The nucleotide and amino acid sequences were compared with the corresponding sequences from susceptible strain *M*. *tuberculosis* H37Rv by using Clustal Omega as described previously [22, 31].

## Results

### Study population and *M*. *tuberculosis* isolates

A total of 256 *M*. *tuberculosis* isolates collected from 256 newly diagnosed TB patients during October 2017 to March 2018 at KNTCL (representing all available isolates during this period) were studied. Most (191 of 256, 74.6%) patients had pulmonary TB. Lack of a proper registry system did not allow us to have complete information for all TB cases. Gender information was available for 251 cases and included 145 (57.8%) male patients. The age of patients ranged from 1 month to 81 years (mean age = 38 ± 13.2 years). The nationality data were available for only 212 patients and included 22 Kuwaiti patients and 190 (89.6%) expatriates. Most expatriates originated from TB endemic countries, particularly from India (n = 66), Philippines (n = 51), Nepal (n = 16), Pakistan (n = 13), Ethiopia (n = 13), Bangladesh (n = 12) and Egypt (n = 5). The date of arrival of expatriate patients in Kuwait was not available.

### Spoligotyping data analysis

**Fig 1:**
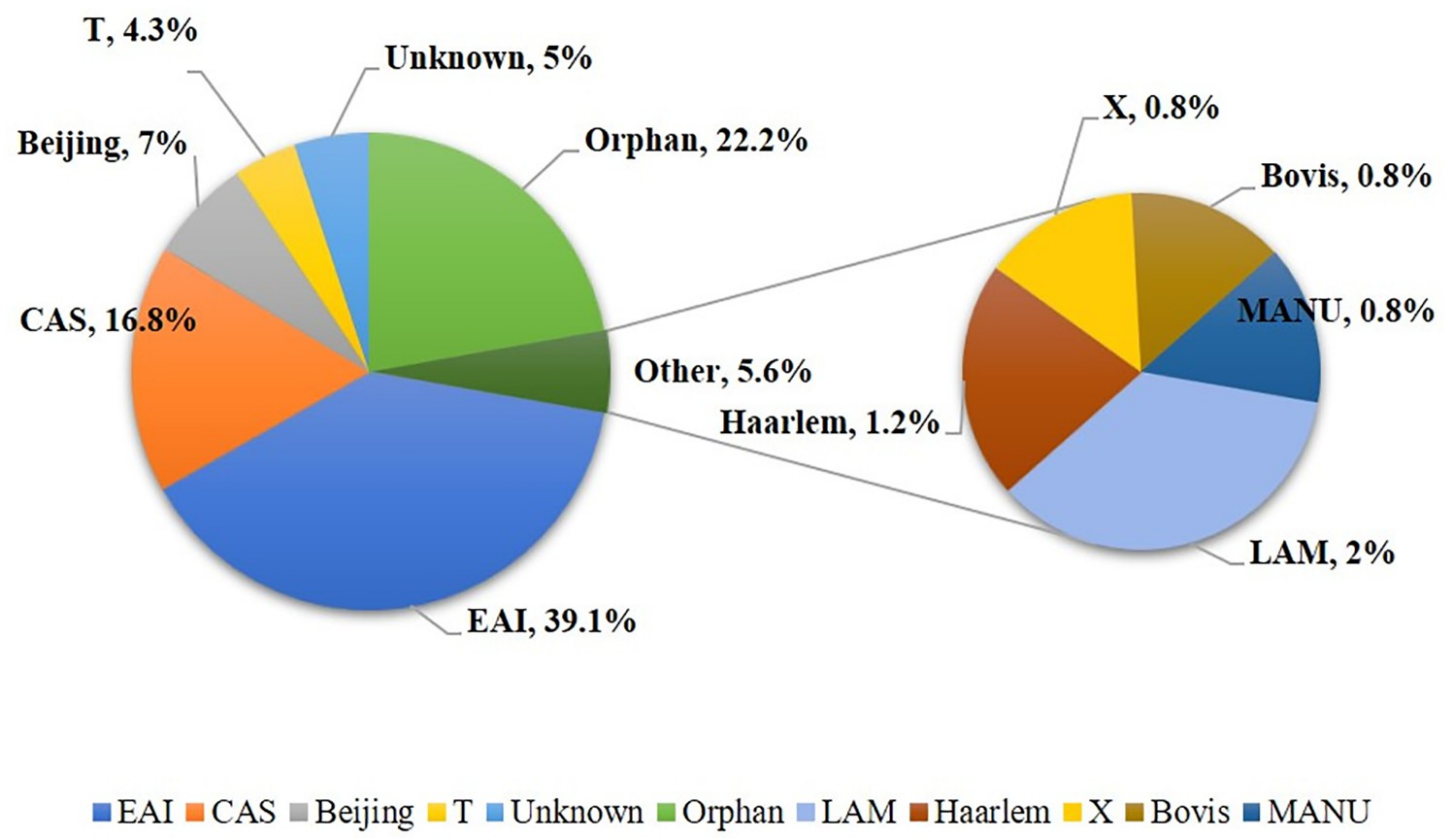
Pie chart showing the distribution of *M*. *tuberculosis* lineages (in percentages) prevalent in Kuwait on the basis of SITVIT2 WEB analysis. EAI, CAS and Beijing lineages were dominant.

The spoligotyping successfully assigned 188 of 256 (73.4%) isolates to specific SITs while, surprisingly, 68 of 256 (26.6%) isolates exhibited new and unique (Orphan) patterns. The *M*. *tuberculosis* isolates belonged to 9 *M*. *tuberculosis* lineages with all major lineages represented among the isolates. Interestingly, three *M*. *tuberculosis* lineages; East African Indian (EAI such as EAI2_Manila), Central Asia Strain (CAS such as CAS1_Delhi) and Beijing lineages were predominant and were represented by 100 of 256 (39.1%), 43 of 256 (16.8%) and 18 of 256 (7.0%) isolates, respectively (Fig 1 and S2 Table). Other less common lineages identified among the isolates included T family (11 of 256, 4.3%), Latin American Mediterranean (LAM; 5 of 256, 2.0%), Haarlem (3 of 256, 1.2%), X family (2 of 256, 0.8%), Bovis (2 of 256, 0.8%) and MANU (2 of 256, 0.8%) family and 57 (22.2%) isolates were identified as “not defined” lineages (Fig 1). Moreover, 13 (5%) *M*. *tuberculosis* isolates (1 isolate with orphan pattern and 12 isolates assigned to eight SITs; SIT27, SIT237, SIT402, SIT1196, SIT1516, SIT1869, SIT1955 and SIT2276) were identified by SITVIT2 database as ‘Unknown’ family. An important observation of our study was the large number (n = 131) of spoligotyping patterns that were seen among 256 isolates including 61 unique and new (orphan) patterns (SITs) shared among 68 isolates (S2 Table). This is particularly striking since the *M*. *tuberculosis* isolates were collected during a short (six-month) period only.

**Fig 2:**
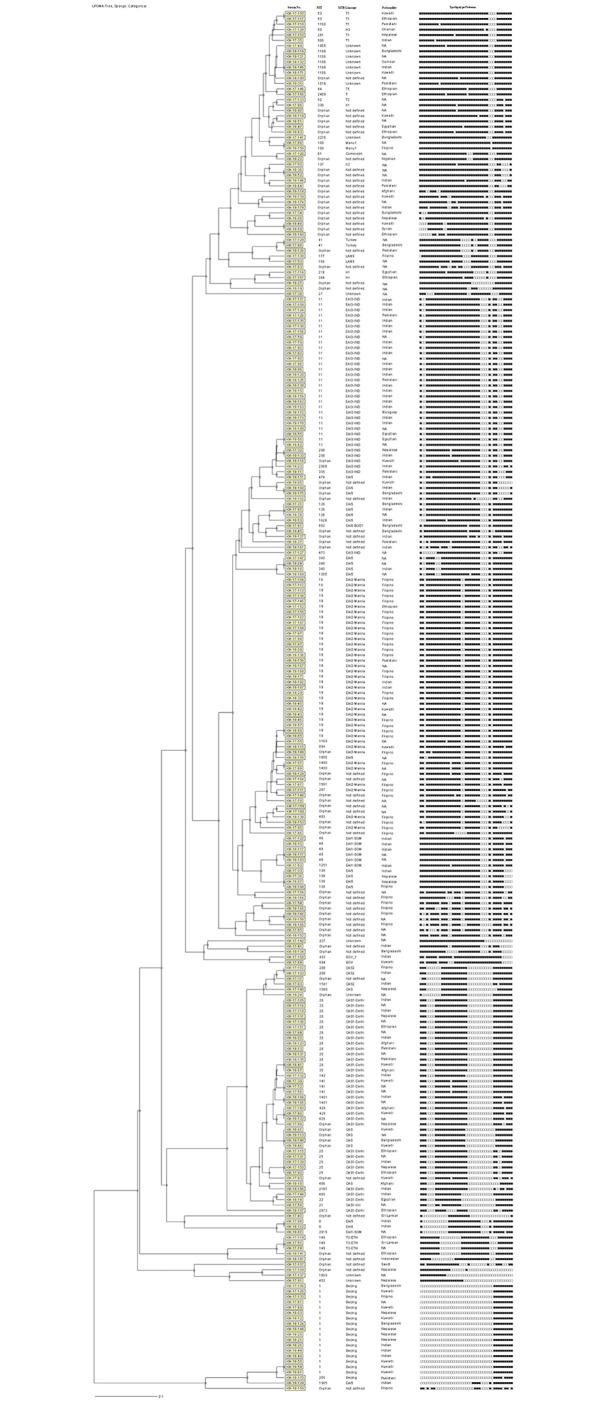
The dendrogram generated from the spoligotyping data using MIRU-VNTRplus database and unweighted pair group method with arithmetic mean (UPGMA) settings for 256 *M*. *tuberculosis* isolates from Kuwait is shown. The shared international type (SIT), *M*. *tuberculosis* family (Family), nationality of the TB patient yielding the isolate and the actual spoligotyping pattern for each isolate are shown in vertical columns.

The dendrogram generated from the spoligotyping data by UPGMA using MIRU-VNTR plus web analysis is shown in Fig 2. The data showed that 105 of 256 (41%) *M*. *tuberculosis* isolates exhibited a unique pattern while 151 of 256 (59%) isolates clustered into 26 SITs (clusters) with each cluster containing 2–30 isolates (clustering rate of 0.488) (Fig 2). The discriminatory power of spoligotyping method for 256 clinical *M*. *tuberculosis* isolates based on HGDI was calculated as 0.966. Of 105 isolates with unique patterns, 56 isolates exhibited a new and unique (orphan) pattern that has not been described previously (Fig 2). Furthermore, 13 *M*. *tuberculosis* isolates (1 isolate with orphan pattern and 12 isolates assigned to eight SITs; SIT27, SIT237, SIT402, SIT1196, SIT1516, SIT1869, SIT1955 and SIT2276) were identified by SITVIT2 database as ‘Unknown’ family.

The largest cluster (SIT19, EAI2_Manila Sub-family) included 30 isolates. Interestingly, 22 of 27 (81.5%) SIT19 isolates (the nationality status of 3 patients was not known) were recovered from Filipino patients. Similarly, the second largest cluster included 28 isolates and 21 of 24 (87.5%) SIT11 isolates (the nationality status of 4 patients was not known) were obtained from patients from South Asia (India, n = 19; Pakistan, n = 2). Although cluster isolates in the SIT1 (Beijing family) were distributed among patients of many nationalities, cluster isolates (n = 14) belonging to SIT26 (CAS1_Delhi Sub-family) were mainly isolated from patients originating from South Asia (India, n = 3, Pakistan, n = 3; Afghanistan, n = 2; Nepal, n = 1) (nationality status of 3 patients was not known) (S2 Table).

The data were also analysed with respect to major *M*. *tuberculosis* lineages detected among patients of the two largest ethnic groups (Indians, n = 66 and Filipinos, n = 51) in Kuwait. Of 66 *M*. *tuberculosis* isolates obtained from Indian patients, 21, 10 and 9 isolates belonged to EAI3-IND, CAS1_Delhi and EAI5 lineages, respectively. Similarly, majority (29 of 51, 56.9%) of isolates from Filipino TB patients belonged to EAI2_Manila lineage while the second largest group included 13 isolates with orphan patterns and undefined *M*. *tuberculosis* lineage (S2 Table).

**Fig 3:**
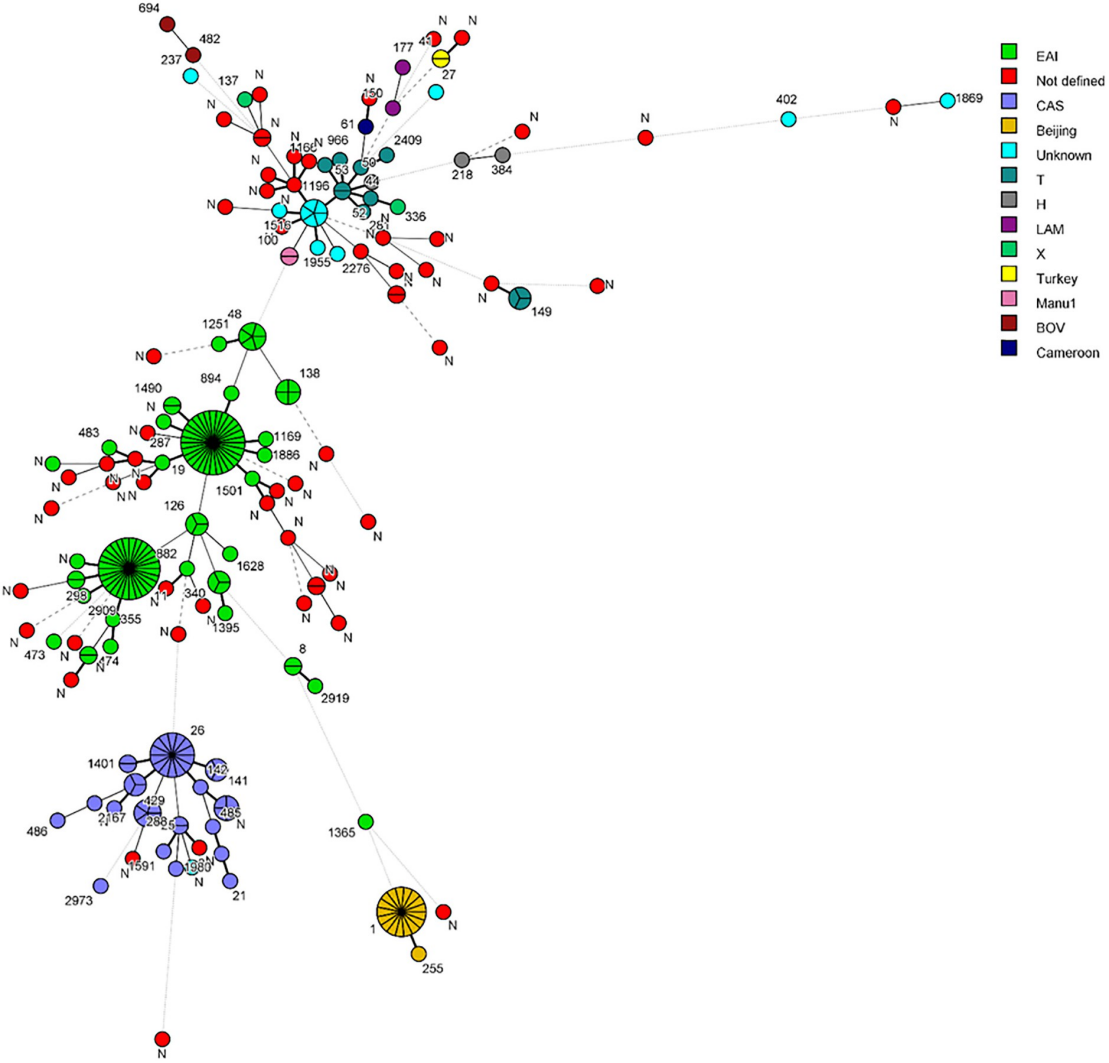
A minimum spanning tree (MST) showing evolutionary relationships based on spoligotypes in *M*. *tuberculosis* population (n = 256 isolates). The phylogenetic tree connects each genotype based on degree of changes required to go from one allele to another. The structure of the tree is represented by branches (continuous vs. dashed and dotted lines) and circles representing each individual pattern. The length of the branches represents the distance between patterns while the complexity of the lines (continuous, gray dashed and gray dotted) denotes the number of allele/spacer changes between two patterns: solid lines, 1 or 2 or 3 changes (thicker ones indicate a single change, while the thinner ones indicate 2 or 3 changes); gray dashed lines represent 4 changes; and gray dotted lines represent 5 or more changes. The size of the circle is proportional to the total number of isolates in the study, presenting unique isolates (smaller nodes) versus clustered isolates (bigger nodes). The color of the circles indicates the phylogenetic lineage to which the specific pattern belongs. The labels of nodes indicate SITs in the study. (N indicates orphan or new spoligotype pattern).

The evolutionary relationships based on degree of changes in spacer patterns that resulted in different alleles among *M*. *tuberculosis* isolates were determined by minimum spanning tree and the results are shown in Fig 3. The data are consistent with the UPGMA-derived tree as the three most dominant *M*. *tuberculosis* lineages in this study were EAI, CAS and Beijing lineages and the most frequently observed patterns were SIT11, SIT19, SIT1 and SIT26, each generating a clonal complex. However, most of the isolates with unique (Orphan) patterns were derived from three clonal complexes originating from EAI2-Manila and EAI3-IND families or an unknown family (represented by SIT1196).

### Drug susceptibility testing of *M*. *tuberculosis* isolates by conventional MGIT 960 system and by GenoType MTBDR*plus* and REBA MTB-MDR assays

The phenotypic DST results against INH, RIF, STR and EMB by MGIT 960 system showed that 241 of 256 isolates were pan-susceptible while 15 isolates were resistant to one or more anti-TB drugs (Table 1). Of 15 drug-resistant isolates, 12 and 5 isolates were INH-resistant and RIF-resistant, respectively. All RIF-resistant isolates were also resistant to INH (MDR-TB strains). The gMTBDR^+^ assay correctly identified susceptibility/resistance to INH and RIF in 14 of 14 pansusceptible isolates that were tested and 10 of 12 INH-resistant and 4 of 5 RIF-resistant isolates (Table 1). Two INH-resistant and 1 RIF-resistant isolate yielded false susceptibility results. All 10 INH-resistant isolates contained S315T mutation in *katG* including 1 isolate that also contained -15 C/T mutation in *inhA* regulatory region (Table 1). DNA sequencing data showed that 2 INH-resistant isolates contained wild-type sequence of *katG* codon 315 region and *inhA* regulatory region. Three of 5 RIF-resistant isolates contained S450L mutation in *rpoB* gene while 1 isolate was identified as RIF-resistant by lack of hybridization with a wild-type probe (Δ*rpoB*WT7) only. DNA sequencing identified R448K mutation in this isolate. One RIF-resistant (additionally resistant to INH, MDR-TB) isolate showed wild-type sequence of *rpoB* hot-spot region and the N-terminal region that are commonly mutated in RIF-resistant strains. Thus, gMTBDR^+^ assay correctly identified only 3 of 5 phenotypically identified MDR-TB strains while 1 isolate each was identified as RIF-monoresistant and INH-monoresistant only. REBA assay correctly identified susceptibility/resistance to INH and RIF in 14 of 14 pansusceptible isolates that were tested and 11 of 12 INH-resistant isolates including 1 isolate (additionally resistant to RIF, MDR-TB) as INH-resistant due to a mutation in *ahpC* promotor region (Table 1). Like gMTBDR^+^ assay, REBA also correctly identified 4 of 5 RIF-resistant isolates including 3 isolates that contained S450L mutation and 1 isolate with R448K mutation (identified by lack of hybridization with *rpoB*WT4 probe in REBA) in *rpoB* gene (Table 1). However, REBA identified MDR status of 4 of 5 MDR-TB strains including 1 isolate that contained a mutation in the upstream region of *ahpC*.

**Table 1:** Phenotypic and genotypic drug susceptibility testing of *M*. *tuberculosis* isolates by MGIT 960 system, MolecuTech REBA MTB-MDR and GenoType MTBDR*plus* assays.

No. of isolates^a^	Phenotypic resistance to anti-TB drugs	Mutation detected by REBA MTB-MDR assay in				Mutation detected by gMTBDR^+^ assay in		
		*rpoB*	*katG315*	*inhA*	*ahpC*	*rpoB*	*katG315*	*inhA*
14	None	WT	WT	WT	WT	WT	WT	WT
3	STR	WT	WT	WT	WT	WT	WT	WT
1	INH	WT	WT	WT	WT	WT	WT	WT
3	INH	WT	S315T	WT	WT	WT	S315T	WT
2	STR, INH	WT	S315T	WT	WT	WT	S315T	WT
1	STR, INH	WT	S315T	-15 C/T	WT	WT	S315T	-15 C/T
1	INH, RIF	Δ*rpoB*WT4^b^	WT	WT	Δ*ahpC*WT1	Δ*rpoB*WT7^b^	WT	WT
1	INH, RIF	WT^c^	S315T	WT	WT	WT^c^	S315T	WT
2	INH, RIF, STR	S450L	S315T	WT	WT	S450L	S315T	WT
1	INH, RIF, STR	S450L	S315T	WT	Δ*ahpC*WT1^d^	S450L	S315T	WT

WT, wild-type; INH, isoniazid; RIF, rifampicin; STR, streptomycin

^a^The remaining drug-susceptible *M*. *tuberculosis* isolates (n = 227) were not analysed by REBA and gMTBDR^+^ assays

^b^PCR sequencing of hot-spot region of *rpoB* identified R448K mutation.

^c^PCR sequencing of hot-spot and N-terminal regions of *rpoB* showed wild-type sequence.

^d^Δ*ahpC*WT1; PCR sequencing identified -43 A/G mutation in the *oxyR*–*ahpC* intergenic region.

## Discussion

The total population of 4.42 million inhabitants in Kuwait in 2018 included 1.34 million (30.2%) Kuwaiti nationals and 3.08 million (69.8%) expatriates (https://www.csb.gov.kw/Pages/Statistics_en?ID=67&ParentCatID=1 accessed on July 14, 2021). The expatriate population is highly diverse but mostly included Indians (22%), Egyptians (15%), Bangladeshis (6%), Filipinos (5%), Syrians (3%), Saudi nationals (3%), Pakistanis (2%), Sri Lankans (2%) and Nepalese (1%). Hence, most of the expatriate population in Kuwait originates from TB endemic countries [4]. It is also pertinent to mention here that all expatriates entering Kuwait are screened and residence visa is granted only to individuals with no evidence of active TB disease. Despite these measures, previous studies have shown that >80% of *M*. *tuberculosis* isolates and >90% of MDR-TB strains are isolated from expatriate TB patients in Kuwait [4, 26, 33]. Fingerprinting of *M*. *tuberculosis* isolates (n = 256) collected from 256 TB patients over a six-month period (October 2017 to March 2018) was performed in this study. Consistent with previously reported data [4, 22, 26], only 22 of 212 (10.4%) subjects were Kuwaiti nationals (country of origin for 44 TB patients was not available) while the remaining (89.6%) patients were expatriates mainly originating from TB endemic countries, particularly from India (n = 66), Philippines (n = 51), Nepal (n = 16), Pakistan (n = 13), Ethiopia (n = 13), Bangladesh (n = 12), Egypt (n = 5) and others (n = 36).

Although spoligotyping exhibits a low discriminatory power and, therefore, is not considered suitable for tracking recent transmission of infection [10, 13], our data revealed high genetic diversity and presence of different *M*. *tuberculosis* families, particularly EAI (EAI2_Manila, EAI3_IND, EAI5, EAI1_SOM & EAI1_BGD1; 39.1%), CAS (CAS, CAS_Delhi, CAS1_Kili & CAS2; 16.8%) and Beijing (7.0%) families represented by different SITs among *M*. *tuberculosis* isolates in Kuwait. The HGDI is the most commonly used estimator of the discriminatory power of a typing method. Although it may overestimate diversity, values in the range of 0.90 to 0.99 have recently been suggested to represent acceptable resolution [34]. The Adjusted Hunter Gaston Index (AHGI) has been derived from HGDI and is projected to give a more realistic discriminatory ability of a typing scheme [34]. Other indices of diversity such as Shannon Index are more suitable for ecological data [34]. An interesting observation of our study was the higher discriminatory power of the spoligotyping method (HGDI = 0.96 and AHGI = 0.82) and the number of different genotypes detected in this study compared to reports from other countries in the region [3, 13, 35–37]. Thus, a large number (n = 131) of spoligotyping patterns were seen among 256 isolates including 61 unique and new (orphan) patterns (SITs) shared among 68 isolates. This is particularly striking since *M*. *tuberculosis* isolates were collected during a short (six-month) period only.

The fingerprinting data showed that 151 of 256 (59%) isolates clustered into 26 SITs (clusters) while 105 isolates (including all 56 isolates with orphan patterns) yielded unique patterns highlighting the presence of a large number of genotypes in Kuwait which are yet to be identified. Majority of orphan patterns were obtained from patients originating from high TB incidence countries (India, n = 9; Philippines, n = 15; Bangladesh, n = 5; Nepal, n = 4; Pakistan, n = 3; and Ethiopia, n = 3) which is likely due to limited spoligotyping studies carried out from different locations in these countries [38–44]. The largest clusters belonged to SIT19 (EAI2_Manilla) shared by 30 isolates, SIT11 (EAI3_IND) shared by 28 isolates, SIT-1 (Beijing) shared by 17 isolates and SIT26 (CAS1_Delhi) shared by 14 isolates. This is consistent with observations showing that >80% of TB patients in Kuwait are expatriates originating mainly from India, Philippines, Nepal, Pakistan and Bangladesh where these lineages are more common [4, 40, 45–48]. The spoligotype pattern SIT19 (EAI2_Manila) is the designated clade for Manila family and is shared by 59% of the EAI strains from Philippines based on SITVIT2 database [40, 45–48]. Consistent with this observation, 22 of 27 (81.5%) SIT19 isolates (the nationality status of 3 patients was not known) in this study were recovered from Filipino patients. Similarly, the second largest cluster in this study (SIT11 or EAI3_IND) is also known to be largely restricted for the Indian sub-continent [38, 48, 49] and 21 of 24 (87.5%) SIT11 isolates (the nationality status of 4 patients was not known) were obtained from patients of south Asian countries. Similarly, most (9 of 11, 81.8%) cluster isolates (nationality status of 3 patients was not known) belonging to SIT26 (CAS1_Delhi Sub-family) were also isolated from patients originating from south Asian countries which is consistent with global epidemiological data [40, 45–47].

The Beijing genotype was identified among both Kuwaiti nationals and non-Kuwaiti expatriate patients in this study with nearly similar frequency. The Beijing genotype originated from China, Korea and Japan and spread through industrialization and urbanization in the twentieth century [50]. It has been reported globally including India, Philippines and Bangladesh whose nationals are major ethnic groups among expatriates in Kuwait and other adjoining Gulf Cooperation Council (GCC) countries [13, 36, 50, 51]. Similar results have also been reported from Saudi Arabia and Oman which also have large expatriate population originating from these countries [13, 51–53]. On the contrary, these lineages (except CAS1_Delhi) are underrepresented in other adjoining countries such as Iraq and Iran which do not have large expatriate population from these countries [3, 36].

Our fingerprinting data were also analysed with respect to major *M*. *tuberculosis* lineages detected among patients of the two largest ethnic groups (Indians, n = 66; Filipinos, n = 51) in Kuwait. Of 66 *M*. *tuberculosis* isolates obtained from Indian patients, 21, 10 and 8 isolates belonged to EAI3_IND, CAS1_Delhi and EAI5 lineages, respectively. Similarly, majority (27 of 51, 52.9%) of isolates from Filipino TB patients belonged to EAI2_Manila lineage while the second largest group included 13 isolates with orphan patterns and undefined *M*. *tuberculosis* lineage. These observations are consistent with the preponderance of these specific lineages among TB patients in India and Philippines [38, 40, 46–49]. Other *M*. *tuberculosis* families identified in Kuwait but in lesser frequency were T, LAM, Haarlem, X, and Manu families which all belong to lineage 4. Interestingly, the three predominant SITs (SIT149, SIT53 and SIT25) reported from Ethiopia [39] were detected among Ethiopian TB patients in Kuwait. Lineage 4 isolates are typically found at high frequencies in all inhabited continents with a predominance throughout Africa, Europe and the region bordering the Mediterranean Sea [54]. Their occurrence in smaller numbers reflect the ethnic fabric of the total population in Kuwait.

The clustering rate in our study was also relatively lower (48.8%) compared to the rates of 86.4% reported from Saudi Arabia [53] and 82.2% reported from Iraq [55]. Thus, the highly variable spoligotyping patterns detected in this study and the fact that vast majority of TB patients were expatriates suggest that most expatriate patients were infected with unique *M*. *tuberculosis* strains likely acquired in their native countries several years ago. These findings are also consistent with previous observations with most drug-resistant *M*. *tuberculosis* strains from Kuwait which have also yielded highly variable genotyping patterns [22, 24, 27]. Although multi-locus sequence analysis in combination with spoligotyping for MDR-TB strains in a previous study from Kuwait suggested transmission of infection from some expatriates to Kuwaiti nationals, the findings could not be confirmed due to incomplete epidemiological link/contact tracing [22]. Similarly, the possibility that some spoligotyping cluster patterns reported in this study might have resulted from recent transmission of infection within Kuwait could not be ruled out as spoligotyping is known to overestimate clustering of isolates [3, 10, 52] and should be combined with data from other genotyping methods (RFLP, 24-loci-based MIRU-VNTR or WGS) for this purpose. Furthermore, patient data registry system needed to trace the source of infection was not available for this study.

We also performed phenotypic DST against four (INH, RIF, STR and EMB) anti-TB drugs by MGIT 960 system and evaluated the performance of REBA assay in comparison with gMTBDR^+^ assay for genotypic detection of resistance to INH and RIF for rapid diagnosis of MDR-TB. Our phenotypic data showed that 15 (5.9%), 12 (4.7%), 5 (2%) and 5 (2%) isolates were resistant to any drug, INH, RIF, and INH + RIF (MDR-TB), respectively. While resistance rates to any drug and INH were lower, resistance rates of nearly 2% for RIF and MDR-TB were slightly higher than annual resistance rates of nearly 1% in previously published reports [4, 26]. These differences could result from the shorter (6-month) duration of the present study. Consistent with previous studies [19, 20], both gMTBDR^+^ and REBA correctly identified susceptibility to INH and RIF in all 14 pansusceptible isolates that were tested indicating very high (100%) specificity of the two tests for the detection of susceptibility to INH and RIF. Furthermore, both, gMTBDR^+^ and REBA correctly identified resistance to RIF in 4 of 5 RIF-resistant isolates. However, REBA performed slightly better than gMTBDR^+^ for the detection of resistance to INH (11 of 12 versus 10 of 12) and MDR-TB (4 of 5 versus 3 of 5) solely due to inclusion of probes corresponding to the upstream region of *ahpC* (*oxyR-ahpC* intergenic region) in the former test. On the contrary, a previous study [20] reported better performance of gMTBDR^+^ (sensitivity of 90.2% and specificity of 98.5%) than REBA (sensitivity of 72.4% and specificity of 98%) for the detection of RIF resistance. It is pertinent to mention here that mutations in *katG* that affect catalase-peroxidase activity cause upregulation of *ahpC* due to compensatory mutations in the *oxyR-ahpC* intergenic region [56, 57]. Mutations in the upstream region of *ahpC* do not directly confer resistance to INH but rather increase the expression of *ahpC* gene and serve as a surrogate marker for INH resistance [56–59]. Thus, occurrence of *M*. *tuberculosis* in samples from locations where *ahpC* mutations are more common has been shown to affect the sensitivity of detection of resistance to INH and MDR-TB [19, 20, 60]. Taken together, our findings show that REBA can also serve as an alternative to gMTBDR^+^ test for the rapid detection of resistance of *M*. *tuberculosis* to RIF, INH and MDR-TB.

Our study has several limitations. i) Not all *M*. *tuberculosis* strains cultured during the six-month period were analyzed in the study as some isolates were lost during storage. ii) Lack of a proper registry system did not allow us to have complete information for all TB cases. As a result, the nationality status of 44 TB patients was not known. iii) The fingerprinting of *M*. *tuberculosis* isolates was performed by spoligotyping only which exhibits a low discriminatory power and is not suitable for tracking recent transmission of infection. iv) Cases of local transmission of TB within Kuwait could not be determined due to lack of patient registry system and epidemiological link data. v) The 24-loci-based MIRU-VNTR typing or WGS was not performed for cluster isolates to rule out recent transmission of infection in Kuwait. vi) The sensitivity and specificity of REBA test for the detection of INH-resistant, RIF-resistant and MDR-TB strains in Kuwait could not be accurately determined due to small number of drug-resistant strains analyzed in this study.

## Conclusion

Molecular fingerprinting of 256 *M*. *tuberculosis* strains collected during a 6-month period mostly from expatriate TB patients in Kuwait by spoligotyping showed high discriminatory power (0.96) reflecting the diversity of *M*. *tuberculosis* SITs in Kuwait. Altogether 131 spoligotyping patterns were seen among 256 isolates including 61 unique and new (orphan) patterns (SITs) shared among 68 isolates. Interestingly, 105 (41%) isolates yielded unique patterns while 151 (59%) isolates clustered into 26 SITs (clusters). The diversity of spoligotyping patterns detected in this study and the fact that vast majority of TB patients were expatriates originating from several TB endemic countries suggest that most expatriate patients were infected with unique *M*. *tuberculosis* strains likely acquired in their native countries several years ago. Our study also showed that REBA MTB-MDR assay is another cost-effective and reliable diagnostic test for rapid molecular detection of resistance of *M*. *tuberculosis* to RIF, INH and MDR-TB.

## Supporting information

S1 Table Clinical source of 256 *M*. *tuberculosis* isolates analyzed by spoligotyping. (DOCX)

S2 Table Clusters and unique *M*. *tuberculosis* patterns among 256 isolates based on spoligotyping data. (DOCX)
