# Supplementary material for: First insights into the phylogenetic diversity of Mycobacterium tuberculosis in Kuwait and evaluation of REBA MTB-MDR assay for rapid detection of MDR-TB
Source: PLoS One. 2022 Oct 20;17(10):e0276487. doi: 10.1371/journal.pone.0276487 (PMC9584360; doi:10.1371/journal.pone.0276487)
Supplement: S1 Table — (DOCX) [file pone.0276487.s001.docx]

**S1 Table**. Clinical source of 256 *M. tuberculosis* isolates analyzed by spoligotyping

| **Clinical specimens** | **No. of *M. tuberculosis* isolates** |
| --- | --- |
| **Pulmonary samples** |  |
| Sputum | 183 |
| Bronchoalveolar lavage (BAL) | 16 |
| Endotracheal tube secretion | 5 |
| Pleural fluid | 5 |
| **Extrapulmonary samples** |  |
| Pus | 18 |
| Fine needle aspirate | 17 |
| Lymph node | 5 |
| Ascitic fluid | 2 |
| Aspiration fluid | 2 |
| Brain mass | 1 |
| Gastric fluid | 1 |
| Stool | 1 |
