# Supplementary material for: First insights into the phylogenetic diversity of Mycobacterium tuberculosis in Kuwait and evaluation of REBA MTB-MDR assay for rapid detection of MDR-TB
Source: PLoS One. 2022 Oct 20;17(10):e0276487. doi: 10.1371/journal.pone.0276487 (PMC9584360; doi:10.1371/journal.pone.0276487)
Supplement: S2 Table — (DOCX) [file pone.0276487.s002.docx]

**S2 Table. Cluster and unique patterns among 256 *M. tuberculosis* (Mtb) isolates based on spoligotyping data**

| **Pattern No.** | **Family** | **SIT** | **Spoligotype Patterns** | **No. of Mtb isolates** | **Nationality** | **Total No. of Mtb isolates** |
| --- | --- | --- | --- | --- | --- | --- |
| 1 | EAI2_Manila | 19 | **1101111111111111111001111111000010111111111** | **22** | Filipino | 30 |
|  |  |  |  | **2** | Indian |  |
|  |  |  |  | **1** | Ethiopian |  |
|  |  |  |  | **1** | Kuwaiti |  |
|  |  |  |  | **1** | Pakistani |  |
|  |  |  |  | **3** | NA |  |
| 2 | EAI2_Manila | 1490 | **1101111111111101111001111111000010111111111** | **1** | Filipino | 2 |
|  |  |  |  | **1** | Indian |  |
| 3 | EAI2_Manila | 287 | **1101111111111111111001111111000010111111011** | **1** | Filipino | 1 |
| 4 | EAI2_Manila | 483 | **1101111111111111111001111111000010111110001** | **1** | Filipino | 1 |
| 5 | EAI2_Manila | 894 | **1101111111111111111001111111000010111110111** | **1** | Kuwaiti | 1 |
| 6 | EAI2_Manila | 1169 | **1101111111110111111001111111000010111111111** | **1** | Filipino | 1 |
| 7 | EAI2_Manila | 1501 | **1101111111111111111001111111000010111101111** | **1** | Filipino | 1 |
| 8 | EAI2_Manila | Orphan | **1101111111111111111001111111000010110000001** | **1** | Filipino | 1 |
| 9 | EAI2_Manila | Orphan | **1101101111111111111001111111000010111111111** | **1** | Filipino | 1 |
| 10 | EAI3_IND | 11 | **1001111111111111111111111111000010110001111** | **19** | Indian | 28 |
|  |  |  |  | **2** | Egyptian |  |
|  |  |  |  | **2** | Pakistani |  |
|  |  |  |  | **1** | Malagasy |  |
|  |  |  |  | **4** | NA |  |
| 11 | EAI3_IND | 298 | **1001111111111101111111111111000010110001111** | **1** | Nepalese | 2 |
|  |  |  |  | **1** | Indian |  |
| 12 | EAI3_IND | 2909 | **1001111111111111111111111111000010110001101** | **1** | Indian | 1 |
| 13 | EAI3_IND | 355 | **1001111111111111111111111111000010110000111** | **1** | Pakistani | 1 |
| 14 | EAI3_IND | Orphan | **1001111111111111110111111111000010110001111** | **1** | Kuwaiti | 1 |
| 15 | EAI3_IND | 473 | **1000000011111111111111111111000010110001111** | **1** | NA | 1 |
| 16 | EAI1_SOM | 48 | **1111111111111111111111111111000010111110111** | **3** | Indian | 5 |
|  |  |  |  | **1** | Filipino |  |
|  |  |  |  | **1** | NA |  |
| 17 | EAI1_SOM | 1251 | **1111111111111101111111111111000010111110111** | **1** | Indian | 1 |
| 18 | EAI1-SOM | 2919 | **1000000000000111111111111111000010111110111** | **1** | Indian | 1 |
| 19 | EAI5 | 138 | **1111111111111111111111111111000010111110000** | **2** | Nepalese | 4 |
|  |  |  |  | **1** | Indian |  |
|  |  |  |  | **1** | Filipino |  |
| 20 | EAI5 | 126 | **1001111111111111111111111111000010111111111** | **1** | Indian | 3 |
|  |  |  |  | **1** | Bangladeshi |  |
|  |  |  |  | **1** | NA |  |
| 21 | EAI5 | 340 | **1001111000111111111111111111000010111111111** | **2** | NA | 3 |
|  |  |  |  | **1** | Indian |  |
| 22 | EAI5 | 8 | **1000000000000111111111111111000010111111111** | **2** | Indian | 2 |
| 23 | EAI5 | Orphan | **1001111111111111111111111111000010110000001** | **1** | Indian | 2 |
|  |  |  |  | **1** | Bangladeshi |  |
| 24 | EAI5 | 474 | **1001101111111111111111111111000010110000111** | **1** | Indian | 1 |
| 25 | EAI5 | 1365 | **0000000000000000000000001111000010111111111** | **1** | Indian | 1 |
| 26 | EAI5 | 1395 | **1001111000111111111111101111000010111111111** | **1** | NA | 1 |
| 27 | EAI5 | 1628 | **0001111111110111111111111111000010111111111** | **1** | Indian | 1 |
| 28 | EAI5 | 1886 | **1100111111111111111001111111000010111111111** | **1** | Filipino | 1 |
| 29 | EAI6_BGD1 | 882 | **1011111111111111111111011111000010111111111** | **1** | Bangladeshi | 1 |
| 30 | Beijing | 1 | **0000000000000000000000000000000000111111111** | **6** | Kuwaiti | 17 |
|  |  |  |  | **4** | Nepalese |  |
|  |  |  |  | **3** | Indian |  |
|  |  |  |  | **2** | Bangladeshi |  |
|  |  |  |  | **1** | Filipino |  |
|  |  |  |  | **1** | NA |  |
| 31 |  | 255 | **0000000000000000000000000000000000111101111** | **1** | Pakistani | 1 |
| 32 | CAS1_DELHI | 26 | **1110000111111111111111000000000000111111111** | **3** | Indian | 14 |
|  |  |  |  | **3** | Pakistani |  |
|  |  |  |  | **2** | Afghani |  |
|  |  |  |  | **1** | Ethiopian |  |
|  |  |  |  | **1** | Kuwaiti |  |
|  |  |  |  | **1** | Nepalese |  |
|  |  |  |  | **3** | NA |  |
| 33 | CAS1_DELHI | 25 | **1110000111111111111111000000000000110011111** | **2** | Indian | 5 |
|  |  |  |  | **2** | Ethiopian |  |
|  |  |  |  | **1** | Nepalese |  |
| 34 | CAS1_DELHI | 141 | **1110000111111101111111000000000000111111111** | **1** | Kuwaiti | 3 |
|  |  |  |  | **1** | Indian |  |
|  |  |  |  | **1** | NA |  |
| 35 | CAS1_DELHI | 142 | **1110000111111111111110000000000000111111111** | **1** | Indian | 1 |
| 36 | CAS1_DELHI | 429 | **1110000111111111111111000000000000111110111** | **1** | Kuwaiti | 3 |
|  |  |  |  | **1** | Afghani |  |
|  |  |  |  | **1** | NA |  |
| 37 | CAS1_DELHI | 1401 | **1110000111111111111111000000000000111101111** | **1** | Indian | 2 |
|  |  |  |  | **1** | NA |  |
| 38 | CAS1_DELHI | 22 | **1110000111111111111000000000000000011111111** | **1** | Egyptian | 1 |
| 39 | CAS1_DELHI | 485 | **1110000111111111111000000000000000111111111** | **1** | Indian | 1 |
| 40 | CAS1_DELHI | 2167 | **1110000111111111111111000000000000100110111** | **1** | Indian | 1 |
| 41 | CAS1_DELHI | 2973 | **1110000111110000011111000000000000110011111** | **1** | Ethiopian | 1 |
| 42 | CAS1_Delhi | Orphan | **1110000111111111111111000000000000111100111** | **1** | Nepalese | 1 |
| 43 | CAS1_Kili | 21 | **1110000110111111111000000000000000011111111** | **1** | NA | 1 |
| 44 | CAS | 486 | **1110000111111111111111000000000000000111111** | **1** | Afghani | 1 |
| 45 | CAS | 1980 | **1110000000111111111111000000000000001111111** | **1** | Nepalese | 1 |
| 46 | CAS | Orphan | **1110000111111111111110000000000000011111111** | **2** | Kuwaiti | 4 |
|  |  |  |  | **1** | Indian |  |
|  |  |  |  | **1** | Bangladeshi |  |
| 47 | CAS2 | 288 | **1110000000111111111111000000000000111111111** | **1** | Indian | 2 |
|  |  |  |  | **1** | Filipino |  |
| 48 | CAS2 | 1591 | **1110000000111111111111000000000000110111111** | **1** | Indian | 1 |
| 49 | T1 | 53 | **1111111111111111111111111111111100001111111** | **1** | Ethiopian | 2 |
|  |  |  |  | **1** | Kuwaiti |  |
| 50 | T1 | 281 | **1111111111111111011111111111111100001111111** | **1** | Nepalese | 1 |
| 51 | T1 | 966 | **1111111011111111111111111111111100001111111** | **1** | Indian | 1 |
| 52 | T1 | 1166 | **1111111110111111111111111111111100001111111** | **1** | Pakistani | 1 |
| 53 | T | 2409 | **1111111111110111111111011111111100001111111** | **1** | Ethiopian | 1 |
| 54 | T2 | 52 | **1111111111111111111111111111111100001110111** | **1** | NA | 1 |
| 55 | T3_ETH | 149 | **1111111110000000000111111111111100001111111** | **1** | Sri Lankan | 3 |
|  |  |  |  | **1** | Ethiopian |  |
|  |  |  |  | **1** | NA |  |
| 56 | T5 | 44 | **1111111111111111111111011111111100001111111** | **1** | Ethiopian | 1 |
| 57 | LAM7_TUR | 41 | **1111111111111111111000001001111100001111111** | **1** | Bangladeshi | 2 |
|  |  |  |  | **1** | NA |  |
| 58 | LAM1_CAM | 61 | **1111111111111111111111000111111100001111111** | **1** | Filipino | 1 |
| 59 | LAM9 | 150 | **1111111111111101111100001111111100001111111** | **1** | Filipino | 1 |
| 60 | LAM9 | 177 | **0111111111111111111100001111111100001111111** | **1** | Filipino | 1 |
| 61 | H1 | 218 | **1111111111110111111111111000000100001111111** | **1** | Egyptian | 1 |
| 62 | H1 | 384 | **1111111111111111111101111000000100001111111** | **1** | Ethiopian | 1 |
| 63 | H3 | 50 | **1111111111111111111111111111110100001111111** | **1** | Ghanian | 1 |
| 64 | X1 | 336 | **1111111111111111101111111111111100001110111** | **1** | NA | 1 |
| 65 | X2 | 137 | **1111111111111111101111111111111100001100001** | **1** | Indian | 1 |
| 66 | BOVIS1_BCG | 482 | **1101111101111110111111111111111111111100000** | **1** | Indian | 1 |
| 67 | BOV | 694 | **1101111101111000111111111111111111111100000** | **1** | Kuwaiti | 1 |
| 68 | MANU1 | 100 | **1111111111111111111111111111111110111111111** | **1** | Filipino | 2 |
|  |  |  |  | **1** | Indian |  |
| 69 | Unknown^a^ | 1196 | **1111111111111111111111111111111100011111111** | **1** | Guinean | 5 |
|  |  |  |  | **1** | Bangladeshi |  |
|  |  |  |  | **1** | Indian |  |
|  |  |  |  | **1** | Kuwaiti |  |
|  |  |  |  | **1** | NA |  |
| 70 | Unknown^a^ | 27 | **1110000111111111111111001111111110000111111** | **1** | NA | 1 |
| 71 | Unknown^a^ | 237 | **1111111111111111111111111111110000000000000** | **1** | NA | 1 |
| 72 | Unknown^a^ | 402 | **1111111111111111111100000000000000000000000** | **1** | Nepalese | 1 |
| 73 | Unknown^a^ | 1516 | **1111111110111111111111111111111100011111111** | **1** | Pakistani | 1 |
| 74 | Unknown^a^ | 1869 | **1111111111110000000000000000000000000000000** | **1** | NA | 1 |
| 75 | Unknown^a^ | 1955 | **1111111111110111111111111111111100011111111** | **1** | NA | 1 |
| 76 | Unknown^a^ | 2276 | **1111111111111111111111111111111000111111111** | **1** | Bangladeshi | 1 |
| 77 | Unknown^a^ | Orphan^c^ | **1000000000111111111111000000000000111111111** | **1** | NA | 1 |
| 78 | Not defined^b^ | Orphan^c^ | **0000111111100111111111111111111100011111111** | **1** | Syrian | 2 |
|  |  |  |  | **1** | Kuwaiti |  |
| 79 | Not defined^b^ | Orphan^c^ | **1111111111111111111111111111111100011000011** | **1** | Nepalese | 2 |
|  |  |  |  | **1** | NA |  |
| 80 | Not defined^b^ | Orphan^c^ | **1001011110111001111001111111000010111101101** | **2** | Filipino | 2 |
| 81 | Not defined^b^ | Orphan^c^ | **1000000000000111111111100000000000000000001** | **1** | Sri Lankan | 1 |
| 82 | Not defined^b^ | Orphan^c^ | **1000111111111111111111111111111100011111111** | **1** | Bangladeshi | 1 |
| 83 | Not defined^b^ | Orphan^c^ | **1011111111111111111111011111000010011111111** | **1** | Bangladeshi | 1 |
| 84 | Not defined^b^ | Orphan^c^ | **1111111111110000001111111111000010111100000** | **1** | Bangladeshi | 1 |
| 85 | Not defined^b^ | Orphan^c^ | **0000001101110111111111111111111100011111111** | **1** | Ethiopian | 1 |
| 86 | Not defined^b^ | Orphan^c^ | **1111111110000000000111111111111100011111111** | **1** | Ethiopian | 1 |
| 87 | Not defined^b^ | Orphan^c^ | **1111111111111111011111111111111100011110111** | **1** | Ethiopian | 1 |
| 88 | Not defined^b^ | Orphan^c^ | **1001011110111001111000111111000000111101101** | **1** | Filipino | 1 |
| 89 | Not defined^b^ | Orphan^c^ | **1001111111111111111001111111000010111100001** | **1** | Filipino | 1 |
| 90 | Not defined^b^ | Orphan^c^ | **1101000010111001111001111111000010111101111** | **1** | Filipino | 1 |
| 91 | Not defined^b^ | Orphan^c^ | **1101011000000000000000000111000010111101101** | **1** | Filipino | 1 |
| 92 | Not defined^b^ | Orphan^c^ | **1101111000111001111001111111000010111101101** | **1** | Filipino | 1 |
| 93 | Not defined^b^ | Orphan^c^ | **1101111110111001111001111111000010111101111** | **1** | Filipino | 1 |
| 94 | Not defined^b^ | Orphan^c^ | **1101111111111111000000111111000010111111111** | **1** | Filipino | 1 |
| 95 | Not defined^b^ | Orphan^c^ | **1101111111111101111001111111000010111101111** | **1** | Filipino | 1 |
| 96 | Not defined^b^ | Orphan^c^ | **1101111111111111011001111111000010111111011** | **1** | Filipino | 1 |
| 97 | Not defined^b^ | Orphan^c^ | **1101111111111111111001111100000010111111111** | **1** | Filipino | 1 |
| 98 | Not defined^b^ | Orphan^c^ | **1111111111110000111001111111000010111100011** | **1** | Filipino | 1 |
| 99 | Not defined^b^ | Orphan^c^ | **1001011110111001111111111111000010110001101** | **1** | Indian | 1 |
| 100 | Not defined^b^ | Orphan^c^ | **1001111111111111111111110100000000110001111** | **1** | Indian | 1 |
| 101 | Not defined^b^ | Orphan^c^ | **1011111111111111111111011101000010111101111** | **1** | Indian | 1 |
| 102 | Not defined^b^ | Orphan^c^ | **1111011111111001110111111111111100011101111** | **1** | Indian | 1 |
| 103 | Not defined^b^ | Orphan^c^ | **1111111110111001101100001111111100011101111** | **1** | Indian | 1 |
| 104 | Not defined^b^ | Orphan^c^ | **1111111111111001111111111111000010111000000** | **1** | Indian | 1 |
| 105 | Not defined^b^ | Orphan^c^ | **1111111111111111111110111111111100011100011** | **1** | Indian | 1 |
| 106 | Not defined^b^ | Orphan^c^ | **1001011111111101111111111111000010111101111** | **1** | Pakistani | 1 |
| 107 | Not defined^b^ | Orphan^c^ | **1111111111111111111000001001111100011111111** | **1** | Pakistani | 1 |
| 108 | Not defined^b^ | Orphan^c^ | **1111111111111111111111111111111100011000011** | **1** | Pakistani | 1 |
| 109 | Not defined^b^ | Orphan^c^ | **1001111111111111111111111111000010110000000** | **1** | Kuwaiti | 1 |
| 110 | Not defined^b^ | Orphan^c^ | **1110000111111101111111000000000000110011101** | **1** | Kuwaiti | 1 |
| 111 | Not defined^b^ | Orphan^c^ | **1111111110111001111111111111110100011101111** | **1** | Kuwaiti | 1 |
| 112 | Not defined^b^ | Orphan^c^ | **1111111111111111111111111110111100011110111** | **1** | Kuwaiti | 1 |
| 113 | Not defined^b^ | Orphan^c^ | **1001111111111111111111111111110100011111111** | **1** | Nepalese | 1 |
| 114 | Not defined^b^ | Orphan^c^ | **1111111111111100000001000000000000000000000** | **1** | Nepalese | 1 |
| 115 | Not defined^b^ | Orphan^c^ | **1110011100111111111111111111111100011111111** | **1** | Afghani | 1 |
| 116 | Not defined^b^ | Orphan^c^ | **1111100000000000111111111111111100011111111** | **1** | Indonesian | 1 |
| 117 | Not defined^b^ | Orphan^c^ | **1111111111111111111111000111111100011111111** | **1** | Nigerian | 1 |
| 118 | Not defined^b^ | Orphan^c^ | **1101111111111111110000100000000100011100011** | **1** | Saudi | 1 |
| 119 | Not defined^b^ | Orphan^c^ | **1111111111111111111101111111111100011110111** | **1** | Egyptian | 1 |
| 120 | Not defined^b^ | Orphan^c^ | **0110000000111111111111000000000000111111111** | **1** | NA | 1 |
| 121 | Not defined^b^ | Orphan^c^ | **1101111111110110111001111111000010111110011** | **1** | NA | 1 |
| 122 | Not defined^b^ | Orphan^c^ | **1101111111111111111001111111000010111001111** | **1** | NA | 1 |
| 123 | Not defined^b^ | Orphan^c^ | **1101111111111111111001111111000010111101001** | **1** | NA | 1 |
| 124 | Not defined^b^ | Orphan^c^ | **1101111111111111111001111111000010111111001** | **1** | NA | 1 |
| 125 | Not defined^b^ | Orphan^c^ | **1111011110111001111111111111000010111100111** | **1** | NA | 1 |
| 126 | Not defined^b^ | Orphan^c^ | **1111111111111001101111111111111100011101111** | **1** | NA | 1 |
| 127 | Not defined^b^ | Orphan^c^ | **1111111111111111101111111111111100011110111** | **1** | NA | 1 |
| 128 | Not defined^b^ | Orphan^c^ | **1111111111111111110111111111111100011111111** | **1** | NA | 1 |
| 129 | Not defined^b^ | Orphan^c^ | **1111111111111111111111001110000000111111111** | **1** | NA | 1 |
| 130 | Not defined^b^ | Orphan^c^ | **1111111111111111111111110000000000011111111** | **1** | NA | 1 |
| 131 | Not defined^b^ | Orphan^c^ | **1111111111111111111111111111111100011110111** | **1** | NA | 1 |

^a^The ‘Unknown’ indicates spoligotype patterns with signatures reported in SITVIT2 database that do not belong to any of the major lineages described in the SITVIT2 database

^b^The ‘Not defined’ lineages indicate an spoligotype pattern that is orphan or new and not reported in SITVIT2 database

^c^An ‘Orphan’ spoligotype pattern did not match with any of the patterns reported previously in SITVIT2 database

NA, not available; Mtb, *M*. *tuberculosis*
